# Feasibility of the Machine Learning Network to Diagnose Tympanic Membrane Lesions without Coding Experience

**DOI:** 10.3390/jpm12111855

**Published:** 2022-11-07

**Authors:** Hayoung Byun, Seung Hwan Lee, Tae Hyun Kim, Jaehoon Oh, Jae Ho Chung

**Affiliations:** 1 Department of Otolaryngology & Head and Neck Surgery, College of Medicine, Hanyang University, Seoul 04763, Korea; 2 Machine Learning Research Center for Medical Data, Hanyang University, Seoul 04763, Korea; 3 Department of Computer Science, Hanyang University, Seoul 04763, Korea; 4 Department of Emergency Medicine, College of Medicine, Hanyang University, Seoul 04763, Korea; 5 Department of HY-KIST Bio-Convergence, College of Medicine, Hanyang University, Seoul 04763, Korea

**Keywords:** machine learning, tympanic membrane, middle ear disease, diagnosis, accuracy

## Abstract

A machine learning platform operated without coding knowledge (Teachable machine^®^) has been introduced. The aims of the present study were to assess the performance of the Teachable machine^®^ for diagnosing tympanic membrane lesions. A total of 3024 tympanic membrane images were used to train and validate the diagnostic performance of the network. Tympanic membrane images were labeled as normal, otitis media with effusion (OME), chronic otitis media (COM), and cholesteatoma. According to the complexity of the categorization, Level I refers to normal versus abnormal tympanic membrane; Level II was defined as normal, OME, or COM + cholesteatoma; and Level III distinguishes between all four pathologies. In addition, eighty representative test images were used to assess the performance. Teachable machine^®^ automatically creates a classification network and presents diagnostic performance when images are uploaded. The mean accuracy of the Teachable machine^®^ for classifying tympanic membranes as normal or abnormal (Level I) was 90.1%. For Level II, the mean accuracy was 89.0% and for Level III it was 86.2%. The overall accuracy of the classification of the 80 representative tympanic membrane images was 78.75%, and the hit rates for normal, OME, COM, and cholesteatoma were 95.0%, 70.0%, 90.0%, and 60.0%, respectively. Teachable machine^®^ could successfully generate the diagnostic network for classifying tympanic membrane.

## 1. Introduction

Examination of the tympanic membrane is the first step in the assessment of otitis media or ear diseases. The classical otoscope contains a light and a magnifying lens to illuminate and zoom in on ear structures. Recently, the video otoscope or ear endoscope has extended the capabilities of the traditional otoscope by providing high quality images, and advances in image processing technology allow tympanic membrane images to be digitized and used for documentation or educational purposes.

Although digital otoscopy has expanded the power of ear examination, the diagnostic accuracy of general practitioners for diagnosing ear pathology was only 30 to 67.5%, relative to otolaryngology specialists, due to lack of experience and skill [1]. Especially in low- and middle-income nations, there are very few otology specialists, which makes it difficult to diagnose and treat patients with middle ear disease early on. Therefore, inaccurate diagnosis results in delayed treatment that could result in preventable complications.

The rapid advances in computing technology and machine learning have led to an explosion in the applications of machine learning in healthcare. In particular, artificial intelligence program as an automatic diagnostic tool has been introduced in the field of medical imaging. For example, a machine learning network automatically reads a chest X-ray or interprets pathology slide for various kinds of cancer. Automatic system has the potential to significantly improve the early detection of diseases. In otolaryngology field, machine learning technologies have been implemented by classifying digitized tympanic membrane images to enhance the diagnostic performance of the ear diseases [2,3,4,5]. Detecting tympanic membrane lesions is one of the most popular applications of computer vision, and diverse algorithms have been proposed for classifying tympanic membrane lesions, such as simple perforations, otitis media with effusion, and cholesteatoma, and these have performed robustly using highly sophisticated neural networks [2,3,5,6,7,8].

Diverse artificial intelligence libraries, including TensorFlow, Pythorch, Python, and Keras, have enabled the application of machine learning technology in health care. However, it remains a challenge for physicians to devise machine learning algorithms on their own without the help of data scientists or computer programmers. Recently, several companies have released user-friendly machine leaning platforms, which do not need coding knowledge. Of these, Teachable Machine^®^, by Google, provides an in-browser machine learning model, and it is the easiest tool for those who were not accustomed to coding skill and knowledge [9,10].

The aim of the present study was to assess the performance and feasibility of Teachable Machine^®^ for the tympanic membrane classification.

## 2. Materials and Methods

### 2.1. Acquisition of Tympanic Membrane Images

A database of tympanic membrane images of individuals who visited the otolaryngology department from January 2015 to December 2020 was available for training and validation of the machine learning network. Images of the tympanic membranes were acquired by a Digital Videoscope (ENF-V2, Olympus, Tokyo, Japan) and registered in a Picture Archiving and Communication System, and the images were extracted and saved in JPEG format, with a resolution of 640 × 480.

### 2.2. Image Annotation and Classifications

All tympanic membrane images were reviewed by three otolaryngology specialists. Out of focus or poor-quality images were not used. The remaining images and corresponding medical records, including operational findings and audiologic test results, were classified as normal, otitis media with effusion (OME), chronic otitis media (COM), or cholesteatoma. OME was diagnosed by the endoscopic finding of middle ear effusion, myringotomy findings, or tympanometry results, and COM refers to a perforated tympanic membrane with or without otorrhea. Cholesteatoma was defined by the presence of an attic retraction pocket or bony destruction with cholesteatoma debris.

**Figure 1:**
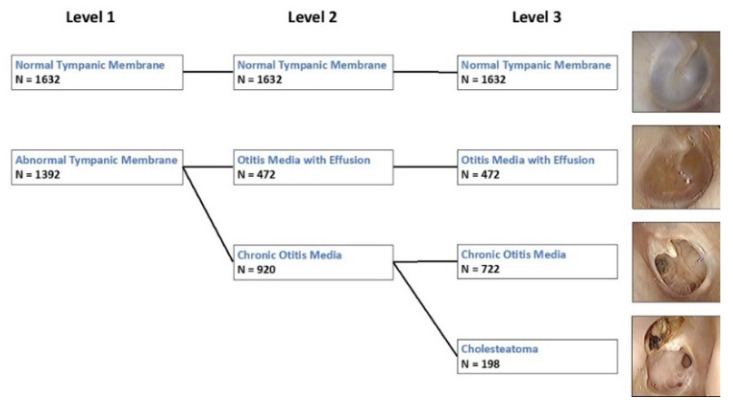
Classification of tympanic membrane abnormalities.

Tympanic membrane image classification was divided into three levels, according to the diagnosis. “Level I” differentiates normal from abnormal tympanic membranes; “Level II” distinguishes between normal, OME, and COM with or without cholesteatoma; and “Level III” differentiates between all four pathologies (normal, OME, COM, and cholesteatoma) (Figure 1). In addition, 20 representative tympanic membrane images for each class, which did not overlap with the training and validation set, were used to evaluate the performance of the devised network. Tympanic membrane image classification was divided into three stages according to the extent of symptom differentiation. “Level I” differentiates normal from abnormal tympanic membranes, “Level II” distinguishes between normal, OME, and COM with or without cholesteatoma, and “Level III” differentiates between all four pathologies (Figure 1). In addition, 20 representative tympanic membrane images for each class, which did not overlap with the training and validation set, were used to evaluate the performance of the devised network.

### 2.3. Teachable Machine

The open artificial intelligence platform of Teachable machine^®^ was used to build the machine learning network for tympanic membrane classification. Teachable machine^®^ builds a pre-training model using a substantial quantity of training data with MobileNet, and the transfer learning is carried out by modifying some of the final layer of the model with uploaded images dataset.

**Figure 2:**
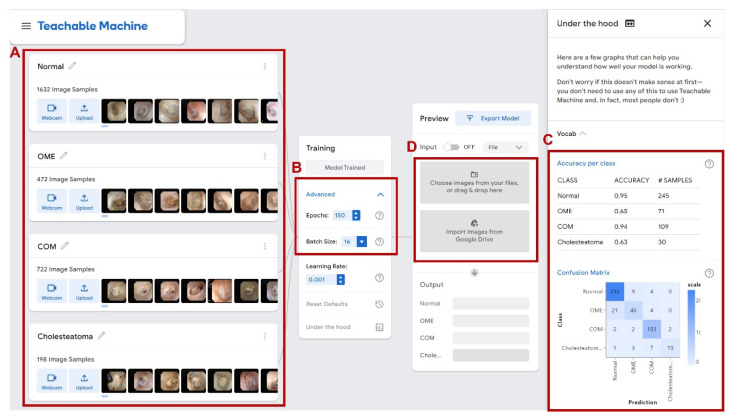
The Teachable Machine^®^ interface. (**A**) Image classification, (**B**) Hyperparameters of epoch and batch size, (**C**) Accuracy per class and confusion matrix with validation set data, (**D**) Input of photo of tympanic membrane to check result of the machine learning network.

At each level, the corresponding tympanic membrane images were uploaded in the system, and the hyperparameters of epoch and batch size were set in the training model. Epoch refers to the number of samples in the training data set that is fed through the training model at least once. In the present study, each level was trained with epoch values of 50, 100, 150, 200, and 250. A batch is the set of samples used in one iteration of training. Batch size as set at 16 (Figure 2). With uploaded tympanic membrane images, 85% of the images were randomly selected and used for the training and the other 15% were used for validation. After training, Teachable machine^®^ generates the diagnostic network and accuracy for each class and confusion matrix for the created model is provided (Figure 2C).

### 2.4. Network Performance and Validation

**Figure 3:**
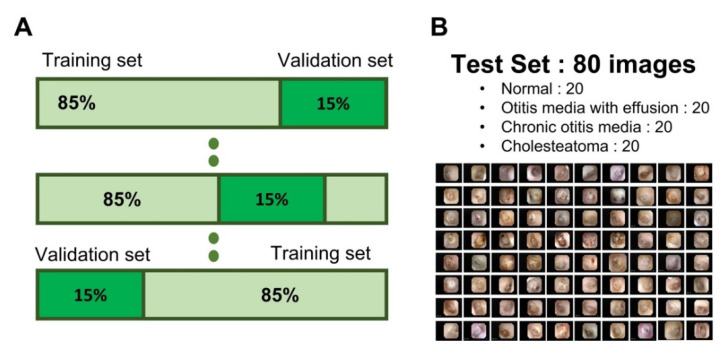
Training, validation, and test set data for tympanic membrane images. (**A**) A total of 85% of the tympanic membrane images were randomly selected and used to train the model, and the remaining 15% were used to check the performance of the model. (**B**) The separate representative tympanic membrane images for testing the performance of the model.

A total of 3024 images was used for training and validation of the machine learning network. At each level, 85% of the images were randomly selected and used for the training and the other 15% were used for validation (Figure 3A). Training and validation were performed 10 times and the corresponding mean accuracy was evaluated at each level. Mann–Whitney test was performed to select the hyperparameter (epoch), which shows the significantly high performance.

To determine the relationship between performance and the number of tympanic membrane images, we evaluated Teachable machine^®^ classification accuracy with 75%, 50%, 25%, and 12.5% of tympanic membrane images at levels I and III.

In addition, the network with highest performance at Level III (to classify all four categories) was selected and assess the performance with 80 representative tympanic membrane images, which did not overlap with the training and validation data sets (Figure 3B).

### 2.5. Ethical Issues

This investigation was approved by the Ethics Review Board of Hanyang University Guri Hospital (IRB # 2022-02-011) and was performed in accordance with the Declaration of Helsinki and good clinical practice guidelines. Informed consent was waived because of the retrospective nature of the study, and the analysis used anonymous clinical data with approval of the ethics review board.

## 3. Results

### 3.1. Tympanic Membrane Images

A total of 3024 tympanic membrane images were used for training and validation. Figure 1 showed the number of annotated tympanic membranes, according to level. In level 1, there were 1632 and 1392 normal and abnormal tympanic membranes, respectively. In Level II, there were 472 abnormal tympanic membrane with otitis media with effusion, and 920 with chronic otitis media with or without cholesteatoma. In the final level, only 722 images with chronic otitis media were distinguished from 198 with cholesteatoma in addition.

### 3.2. Network Verification

**Figure 4:**
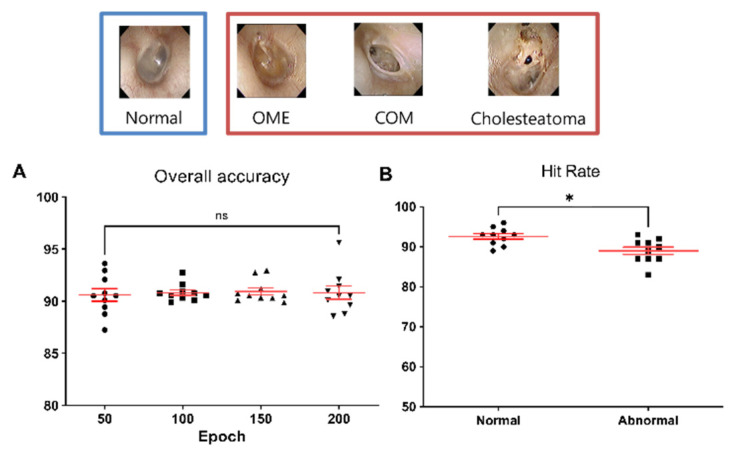
The accuracy and hit rates for differentiating normal versus abnormal tympanic membranes. (**A**) Overall accuracy of the machine learning model, according to epoch. (**B**) Mean hit rates for normal (92.6%) and abnormal (89.0%) tympanic membranes and showed significant difference (* *p* < 0.0087).

Figure 4A indicates the overall accuracy of the network, according to the epoch. The mean accuracy was 90.8 ± 1.5% and the mean hit rates for normal and abnormal were 92.6 ± 2.1% and 89.0 ± 2.7% at the highest performance, and the mean hit rate for normal tympanic membrane was significantly higher than that of abnormal tympanic membrane (*p* = 0.0087) (Figure 4B).

**Figure 5:**
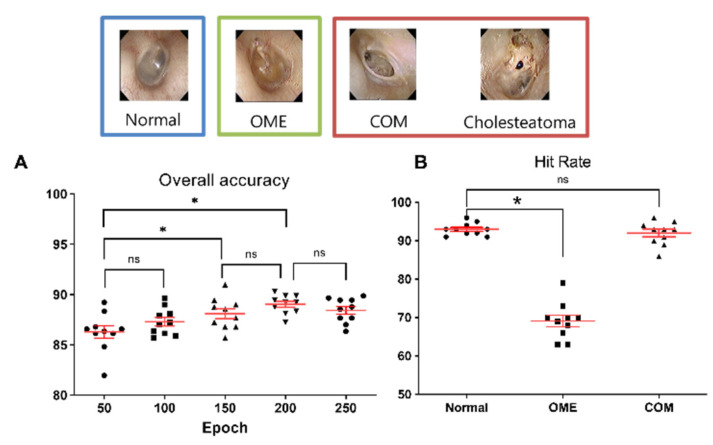
Accuracies and hit rates for differentiating between normal, otitis media with effusion, and chronic otitis media, including cholesteatoma. (**A**) Overall accuracy for the machine learning model, according to epoch. (* indicates *p*-value less than 0.05 with Mann–Whitney test, ns meant non-significant difference). (**B**) Mean hit rates for normal (93.0%), otitis media with effusion (69.1%), and chronic otitis media, including cholesteatoma (92.0 (* indicates *p*-value less than 0.05 with Mann–Whitney test, ns meant non-significant difference).

In Level II, the mean accuracy of the network ranged from 86.3% to 89.0%, according to the epoch, and the epoch value of 200 showed the highest performance (Figure 5A). In addition, hit rates for normal tympanic membrane, OME and COM with/without cholesteatoma were 93.0 ± 1.6%, 69.1 ± 4.5%, and 92.0 ± 2.9% (Figure 5B). Mean hit rate for normal tympanic membrane was not significantly different from that of COM (Figure 5A, *p* = 0.615), however, mean hit rate for OME showed significantly lower value than that of normal tympanic membrane (*p* < 0.001).

**Figure 6:**
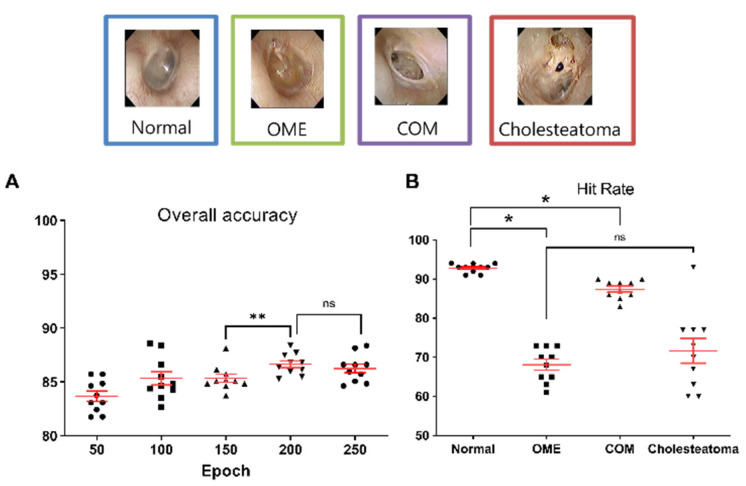
Accuracies and hit rates for differentiating between normal, otitis media with effusion, chronic otitis media, and cholesteatoma. (**A**) Overall accuracies of the machine learning model, according to epoch. (** indicates *p*-value less than 0.05 with Mann–Whitney test, ns meant non-significant difference). (**B**) Mean hit rates for normal (92.8%), otitis media with effusion (68.1%), chronic otitis media (87.4%), and cholesteatoma (71.7%). (* indicates *p*-value less than 0.05 with Mann–Whitney test, ns meant non-significant difference).

In the final stage (Level III), the overall mean accuracy was 85.4 ± 1.7% (Figure 6A) and the average hit rate for normal tympanic membranes was 92.8% ± 1.1%, while hit rates for OME, COM, and cholesteatoma were 68.1% ± 4.8%, 87.4% ± 2.2%, and 71.7% ± 9.6%, respectively (Figure 6B). The mean hit rate for normal tympanic membrane was significantly higher than those of the normal, OME, and cholesteatoma, and the hit rate for OME and cholesteatoma did not show significant difference (*p* = 0.465).

The accuracy for Level I was significantly higher than those for Level II and III, respectively (*p* < 0.001, *p* < 0.001), and the accuracy for Level III was significantly lower than the accuracy in Level II (*p* = 0.002).

### 3.3. Diagnostic Performance According to the Number of Tympanic Membrane Image

**Figure 7:**
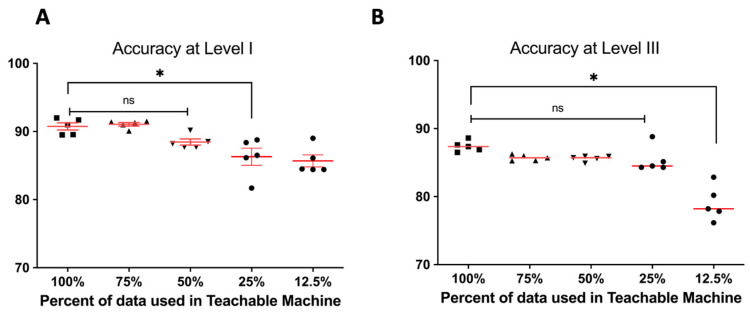
Accuracy of Teachable Machine^®^ according to the amount of data used in training and validation. (**A**) Mean accuracies of model according to the amount of data at Level I. (* indicates *p*-value less than 0.05 with Mann–Whitney test, ns meant non-significant difference). (**B**) Performance of Teachable machine according to the number of images at Level III (* indicates *p*-value less than 0.05 with Mann–Whitney test, ns meant non-significant difference).

The developed network’s accuracy with the entire image data set was highest at Levels I and III. It was confirmed that the accuracy decreased as the number of images used for training decreased both Level I and III. The accuracy was significantly reduced when learning with only 25% of the data at Level I, and the performance deteriorated when learning with 12.5% of the data at Level III (Figure 7).

### 3.4. Performance of the Network with Representative

#### Tympanic Membrane Images

**Figure 8:**
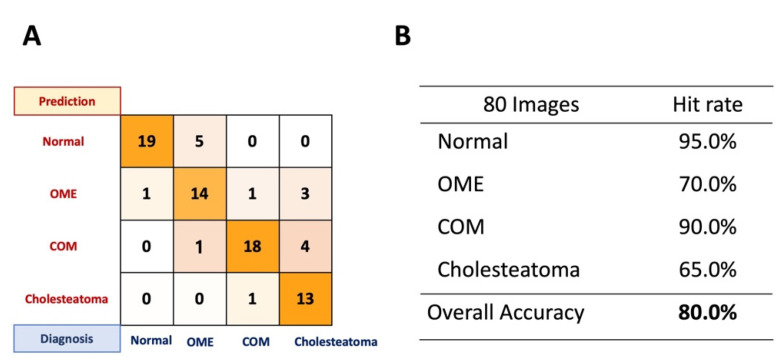
Performance of the machine learning network with 80 representative images. (**A**) Confusion matrix for representative images. (**B**) Overall accuracy of the machine learning network devised with Teachable Machine and hit rates for each classification.

The performance of the devised network with eighty representative tympanic membrane images (20 for each level) achieved an accuracy of 78.75%. Hit rate for identifying normal tympanic membranes was 95.0%; for OME it was 70.0%, for COM it was 90.0%, and for cholesteatoma it was 60% (Figure 8A). Figure 8B shows the confusion matrix for the performance of Teachable Machine. OME cases were often misdiagnosed as normal, and it was hard to distinguish cholesteatoma from COM and OME.

## 4. Discussion

The present study used the non-coding machine learning platform Teachable Machine^®^ to classify tympanic membrane lesions and has assessed the performance of the devised network. The findings of this study are: (1) The mean accuracies of the network for classifying Level I (normal vs. abnormal), II (normal, OME, and COM), and III (normal, OME, COM, and cholesteatoma) were 90.8 ± 1.5%, 87.8 ± 1.7% and 85.4 ± 1.7%, respectively (2) The mean accuracy for classifying representative tympanic membrane images was 78.75%. To the best of our knowledge, the present study is the first to assess the use of a non-coding machine learning platform to classify tympanic membrane lesions. We believe our findings will contribute to the expansion of machine learning-related diagnostic technologies.

Chronic otitis media is the leading cause of hearing loss, especially in developing countries [1]. However, correct otoscopic examination and interpretation still require training and experience, and the accuracy in diagnosing otitis media of primary care physicians was substantially lower than that of otolaryngology specialists [1,11,12]. Therefore, in situations where otolaryngologists are not present, automatic diagnosis of middle ear illness using machine learning models can be very helpful. In this regard, substantial research had been conducted to develop machine learning networks for the diagnosis of tympanic membranes and showed the robust performance. However, in order to implement artificial intelligence technologies in clinical practice, coding or programming skills are required. In the present study, we tested the feasibility of non-coding machine learning platform Teachable Machine^®^ to diagnose tympanic membrane lesion.

The Google Teachable Machine^®^ is a web-based, open-source platform that enables the creation and training of supervised machine learning models without the need for any kind of programming language. It uses transfer learning algorithm and trains and runs the models in web browser using TensorFlow.js. A recent study showed the feasibility of Teachable Machine^®^ to diagnose tooth-marked tongue with 634 tooth-marked tongue images and 491 non-tooth marked, normal tongue images and demonstrated that the accuracy for diagnose tooth-marked tongue was 92.1% [10]. They proposed the applicability of Teachable Machine^®^ in clinical practice [10].

Several studies have leveraged deep learning networks to classify middle ear disease into the binary classification of normal versus abnormal tympanic membrane. The accuracy of previously published machine learning algorithms in binary classification ranged from 76.0% to 94.2% [5,8,13,14,15]. In the present study, the accuracy for classifying normal versus abnormal tympanic membrane was 90.8% and the performance of Teachable machine^®^ to detect abnormal tympanic membrane image was comparable to the previously reported customized networks (Table 1).

In case of multiple classifications, an early machine learning network for classifying tympanic membrane lesions into the five categories of normal, wax, acute otitis media, chronic otitis media, and otitis media with effusion had an average accuracy of 86.84% for images captured with commercial video otoscopes, using 389 images [19]. Wu et al. used 10,703 pediatric tympanic membrane images for training to differentiate normal, OME and acute otitis media, and 1500 images for validation, and showed the performance of their network to be around 90% [4]. Recent machine learning studies have achieved a robust diagnostic accuracy in multiple classification tasks. When differentiating normal tympanic membranes, impacted cerumen, myringosclerosis, and chronic otitis media, a machine learning system using public otoscopic imaging achieved an accuracy of 93.9%, sensitivity of 87.8%, specificity of 95.9%, and positive predictive value of 87.7% [15]. Another study showed that a machine learning network that had been trained with 10,544 otoendoscopy images of normal, perforation, otitis externa, tumors, and attic retraction to diagnose middle ear or external ear conditions had a mean accuracy of 93.67% with an ensemble model of Inception V3 and ResNet101 [15,16].

The current network devised from Teachable machine^®^ had a performance of about 85% in classifying the four kinds of tympanic membrane lesions of normal, OME, COM, and cholesteatoma. By breaking down the diagnostic accuracy of each disease, we found that the network from Teachable machine^®^ successfully identified normal tympanic membranes and perforation (COM) but had difficulty in detecting OME and cholesteatoma. It is interesting to note that resident otolaryngologists also struggled to recognize the difference between a cholesteatoma and a COM, as well as between a normal tympanic membrane and OME [3]. Based on this, we assume that the Teachable machine^®^ did not execute at a professional level in multiple classifications.

To train the machine learning algorithm, it is important to prepare enough good quality medical images. In cases where the image data are insufficient for learning, data augmentation processes, such as cropping, flipping, rotation, and contrast adjustment, can be performed to increase the number of training images [3,20]. Since the teachable machine does not include an image preprocessing function, the performance can only be achieved when there is a sufficient amount of data. The present achieved the comparable performance because of an adequate number (3024) of tympanic membrane images. However, performance was unaffected substantially by halving the number of images used for assessment (Figure 7).

The present study showed that the performance of the network devised by Teachable machine^®^ was similar to that of the early version of machine learning networks for tympanic membrane classification, researchers who want to start medical imaging-related AI research may be able to use Teachable machine^®^ as a tool to evaluate the feasibility of their research approach [10]. When preparing AI research with medical images, Teachable machine^®^ could provide preliminary results and reference data for target performance. In this regard, it will be interesting to test the performance of Teachable machine^®^ by applying it to medical images, such as more complex X-ray or laryngoscope images.

The current study demonstrated that using Teachable machine^®^ for classifying tympanic membrane lesions would be feasible and comparable to previous investigations. However, this study has several limitations. First, since the images used were derived from ear endoscopy in a single institution, the universal applicability of such networks will have to be established by cross-validation with outside tympanic membrane images or images from an open-source data base. Second, although the present study used representative images, many other types of disease were not addressed. We could not include diseases with relatively low prevalence or those that were difficult to see in a tertiary hospital. Impacted cerumen, external otitis, acute otitis media, and malignancy, etc., will have to be included to assure the wider application of the network in real clinical practice.

## 5. Conclusions

The present study has devised a machine learning network for tympanic membrane classification without coding knowledge. Teachable machine^®^ could successfully generate the diagnostic network for classifying tympanic membrane.

## Figures and Tables

**Table 1:** Performance of the tympanic membrane classification algorithm.

Study	Tympanic Membrane Classification	Number of Classification	Algorithm Used	Accuracy
The present study	Normal versus Abnormal	2	Teachable Machine^®^	90.8 ± 1.5%
	Normal, OME, and COM	3	Teachable Machine^®^	87.8 ± 1.7%
	Normal, OME, perforation, cholesteatoma	4	Teachable Machine^®^	85.4 ± 1.7%
Alhudhaif et al. (2021) [7]	Normal, AOM, CSOM, Earwax,	4	CBAM	98.26%
Crowson et al. (2021) [16]	Normal versus OME	2	ResNet34	84.06%
Tsutsumi et al. (2021) [14]	Normal versus abnormal	2	InceptionV3	73.0%
			MobileNetV2	77.0%
Habib et al. (2020) [8]	Normal versus Perforation	2	InceptionV3	76.00%
Cai et al. (2021) [17]	Normal, OME, CSOM	3	Resnet50	93.4%
Wu et al. (2021) [4]	Normal, AOM, OME	3	Xception	90.66%
		3	MobileNetV2	88.56%
Cha et al. (2019) [15]	Normal versus Abnormal	2	InceptionV3	93.31%
		2	ResNet101	91.88%
		2	Ensemble Network	94.17%
Livingstone et al. (2019) [18]	Normal, Earwax, Tympanostomy tube	3	CNN	84.44%

OME; Otitis media with effusion, COM; Chronic otitis media, AOM; Acute otitis media, CSOM; Chronic suppurative otitis media.

## Data Availability

The data presented in this study are available on request from the corresponding author.
